# The Effectiveness of Low-Intensity Psychological Interventions for Comorbid Depression and Anxiety in Patients with Long-Term Conditions: A Real-World Naturalistic Observational Study in IAPT Integrated Care

**DOI:** 10.1007/s12529-023-10215-9

**Published:** 2023-09-11

**Authors:** Chi Tak Lee, Siobhan Harty, Adedeji Adegoke, Jorge Palacios, Claire M. Gillan, Derek Richards

**Affiliations:** 1 School of Psychology, Trinity College Dublin, Dublin, Ireland; 2 Trinity College Institute of Neuroscience, Trinity College Dublin, Dublin, Ireland; 3 SilverCloud Science, SilverCloud Health, Dublin, Ireland; 4 Global Brain Health Institute, Trinity College Dublin, Dublin, Ireland

**Keywords:** Long-term condition, Psychological comorbidities, Depression, Anxiety, Psychological interventions, Real world

## Abstract

**Background:**

Low-intensity psychological interventions may be a cost-effective, accessible solution for treating depression and anxiety in patients with long-term conditions, but evidence from real-world service settings is lacking. This study examined the effectiveness of low-intensity psychological interventions provided in the Improving Access to Psychological Therapies programme in England for patients with and without long-term conditions.

**Methods:**

A retrospective analysis was conducted on patients (total *N* = 21,051, long-term conditions *n* = 4024) enrolled in three low-intensity psychological interventions, i.e. Internet-delivered cognitive behavioural therapy (iCBT), guided self-help (GSH), and psychoeducational group therapy (PGT) within a Talking Therapies service from 2016 to 2020. Primary outcomes included pre–post-treatment changes in depression (Patient Health Questionnaire-9) and anxiety (Generalised Anxiety Disorder-7).

**Results:**

Overall, both cohorts significantly improved on all outcomes post-treatment, with large effect sizes. Patients with long-term conditions experienced a greater reduction in depression while those without experienced a greater reduction in anxiety, but these differences were marginal (< 1 score difference on both measures). No difference between the cohorts was shown when comparing the differential effectiveness across interventions, but those engaging in iCBT showed greater reduction in depression and anxiety than those in GSH and PGT, while those in GSH improved more than PGT.

**Conclusions:**

Low-intensity psychological interventions, particularly iCBT, were effective in treating depression and anxiety in patients with long-term conditions in a real-world service setting. Our large-scale study supports the continued and increased implementation of low-intensity psychological interventions for this subpopulation via integrated care.

**Supplementary Information:**

The online version contains supplementary material available at 10.1007/s12529-023-10215-9.

## Introduction

Common psychological disorders, such as depression and anxiety, are two to three times more likely to occur in people with physical long-term conditions (LTCs), for instance, diabetes, cardiovascular disease, arthritis, and asthma, when compared with the general population [1]. The co-occurrence of these psychological disorders can significantly impact the prognosis for the LTC, as well as the individual’s broader quality of life across different domains [2, 3]. For example, depression and anxiety were significantly associated with poorer health-related (e.g. physical functioning, difficulties with medical care) and work-related outcomes (e.g. more sick leave and work interference) in patients with diabetes [4]. LTC patients with comorbid depression have also been shown to exhibit less effective self-care in the form of poorer treatment adherence for their physical condition (i.e. three times the odds of treatment noncompliance compared to non-depressed patients) [5]. This is not surprising, as the interplay between physical and mental illness has been suggested to raise healthcare costs by at least 45% for each LTC patient experiencing comorbid mental health problems [3], a considerable increase for a population found to disproportionately live in deprived areas with reduced access to all kinds of resources [6]. If left untreated, depression and anxiety can further exacerbate the complications surrounding the LTCs, such that several studies have posited a link between these psychological comorbidities and increased use of urgent care [1, 7] and risk of mortality [8, 9].

Considering the magnitude of complications that arise when depression and anxiety accompany a LTC, it is imperative to effectively address these psychological comorbidities. One way to manage depression and anxiety symptoms in people with LTCs is through cognitive behaviour therapy (CBT) [10]. The fundamental premise of CBT is that thoughts, physical symptoms, mood, and behaviours are all interrelated, and the way people make sense of their environment affects their feelings and behaviours. CBT has been delivered through evidence-based protocols to people with LTCs; the goal is to alleviate their depression and anxiety symptoms so as to reduce the burden these may have on one’s self-management [11]. However, many significant barriers remain for people with LTCs that limit their capacity to benefit from CBT, including difficulty in accessing treatments due to physical limitations imposed by their LTC, increased healthcare costs in managing multiple illnesses, and disjointed, insufficient service delivery in meeting both their physical and mental health needs [3].

In the UK, the Improving Access to Psychological Therapies (IAPT) are working towards an integrated stepped-care model to extend their access to people with LTCs for treating comorbid depression and anxiety symptoms [12]. In line with current NICE recommendations [10, 13], IAPT delivers CBT-based low-intensity psychological interventions to patients with LTCs. These include guided self-help (GSH) programme which combines written self-help materials with telephone support, psychoeducational group therapy (PGT) which delivers psychoeducation in a group-based format, and internet-based cognitive behavioural therapy (iCBT) which is a clinician-guided intervention delivered online. While these interventions typically follow a standardised treatment protocol and may not tailor to the specific needs of individuals presenting with a particular LTC, they require less intensive resources for implementation (i.e. less clinician time and involvement, lowered costs) and can therefore help improve treatment access which is a proven obstacle for LTC patients [12].

The effectiveness of these low-intensity psychological interventions has been widely established in the general patient population. A large-scale naturalistic cohort study of IAPT patients found these interventions to be effective in improving depression, anxiety, and impaired functioning, revealing iCBT to have a greater average treatment effect when compared to GSH and PGT [14]. These interventions also enjoy comparable effectiveness to traditional face-to-face CBT, with several systematic reviews and meta-analyses suggesting no association between varying delivery formats of CBT and outcomes [15–17]. In this regard, however, evidence for their effectiveness in people with LTC is lacking. Firstly, most clinical trials investigating psychological interventions disregard patients with an accompanying physical illness and its impact. Of the existing studies, systematic reviews found preliminary, favourable evidence for the use of low-intensity psychological interventions for patients in LTC, but they also highlighted crucial limitations such that studies are generally underpowered [18, 19] and have poor to fair methodological quality [19–21]. The present study sought to fill this gap, by comparing the effectiveness of low-intensity psychological interventions provided through IAPT for over 4000 LTC patients and over 17,000 non-LTC patients, in a real-world setting. The study additionally examined whether the LTC cohort showed differential response to iCBT, guided self-help, and psychoeducational group therapy when compared to non-LTC patients.

## Methodology

### Study Design and Setting

The study adopted a retrospective, observational design examining four years of routine data of patients within Talking Therapies, an IAPT service in Berkshire Healthcare NHS Foundation Trust. Talking Therapies employs a stepped-care model of psychological care for patients by matching treatment intensity to their needs. Each patient has an initial appointment with a clinician, who assesses the severity of their symptoms so to determine an appropriate care pathway in conjunction with the patient. If deemed suitable by the clinician in agreement with the patient, iCBT, GSH, or PGT is offered as low-intensity intervention as treatment options.

### Participants

The study examined patients experiencing depression and/or anxiety symptoms who were enrolled in iCBT, PGT, or GSH at step 2 or 3 within Talking Therapies from April 1st, 2016, to March 31st, 2020. In line with the IAPT reporting criteria for establishing universally measurable outcomes [12], a course of treatment is defined as attendance at two or more treatment sessions (in-person or via telephone contact) or receiving two or more online reviews (i.e. patients were not required to have completed the entire course of treatment). Therefore, the study excluded data from patients who did not complete a course of treatment, as well as those below 18 years old at the initial assessment appointment. According to the IAPT-LTC service implementation guide, a LTC is defined as a range of long-term physical health conditions including, but not limited to, cardiovascular disease, chronic obstructive pulmonary disease, diabetes, chronic pain, and musculoskeletal disorders [12]. Patients indicated ‘Yes’ or ‘No’ for their LTC status and those who did not provide this information were further excluded from analyses in the study. As a result, 21,051 patients were included in the study, of which 4024 had at least one LTC, defined as (19.1%). It is noteworthy to highlight that information regarding the specific type of LTC(s) each patient had in this study was not available to the authors for analyses.

### IAPT Low-Intensity Interventions

#### Internet-Delivered Cognitive Behavioural Therapy (iCBT)

The iCBT programmes consist of seven online modules following evidence-based CBT principles for the treatment of depression and/or anxiety [22]. The intervention comprises common cognitive, emotional, and behavioural components (e.g. behavioural activation, mood and lifestyle monitoring) and additionally tailors content to the patient’s mental health diagnosis and presentations (e.g. *Challenging Core Beliefs* for depression; *Worry Tree* for anxiety). Programme content includes several forms of rich media content (videos, quizzes, animations, audio) to facilitate the delivery of the intervention. Each patient is assigned a single, trained clinician who guides treatment progress and responds to queries through weekly/bi-weekly reviews. The recommended duration of guided iCBT is 6–8 weeks, after which the patient can still access the programme for up to 12 months.

#### Guided Self-Help (GSH)

GSH begins with a face-to-face consultation between the patient and their clinician for treatment planning. The treatment plan incorporates CBT-based strategies using written self-help materials, which include information on the patient’s specific condition and CBT-based techniques for self-management (e.g. behavioural activation, cognitive restructuring), along with related homework exercises [23]. Clinician support is provided through 4–6 telephone calls typically scheduled every two weeks, each lasting 20–25 min.

#### Psychoeducational Group Therapy (PGT)

Typically facilitated by two clinicians, PGT is a group-based course delivering CBT psychoeducation for managing depression and anxiety symptoms. PGT seeks to normalise patients’ difficulties within a group setting. Patients are encouraged to share their experiences and discuss relevance of taught materials amongst peers to increase awareness of individual issues in a collective manner. Patients are also tasked with small homework exercises, taking 15–20 min daily. The recommended duration of PGT consists of four weekly sessions, each lasting approximately 90 min with up to 15 patients in attendance at once.

### Outcome Measures

This study included depression and anxiety as the primary outcomes and functional impairment as the secondary outcome.

#### Patient Health Questionnaire-9 (PHQ9)

This is a nine-item self-report of depression symptoms with a total score ranging from 0 to 27, where higher scores indicate more severe levels of depression. The PHQ-9 distinguishes well between depressed and non-depressed individuals using the clinical cut-off total score ≥ 10 with good reliability and validity [24].

#### Generalised Anxiety Disorder (GAD-7)

This is a seven-item self-report of anxiety symptoms with a total score ranging from 0–27, where higher scores indicate more severe levels of anxiety. The GAD-7 uses a cut-off point of ≥ 8 and has good convergent validity with anxiety scales and good reliability [25].

#### Work and Social Adjustment (WSAS)

This is a five-item self-report of functional impairment, examining the experiential impact of a disorder across different life domains from the perspective of the patients. The WSAS has a total score ranging from 0 to 40, where higher scores indicate poorer adjustment. The measure has also demonstrated good reliability and sensitivity [26].

### Data Analysis

The study analysed four years of IAPT patient data from April 1st, 2016, to March 31st, 2020. Baseline demographics and symptom severity were compared between LTC and non-LTC cohorts across three low-intensity interventions. Patients’ depression, anxiety, and functional impairment scores in their last treatment session before discharge were treated as their ‘post-treatment’ score. Outcome variables of Recovery, Reliable Improvement, and Reliable Recovery defined according to the IAPT reporting criteria [12] were compared between LTC and non-LTC cohorts. IAPT Reliable Change Indices (RCI) of six and four points were used as cutoffs to measure reliable change on the PHQ-9 and GAD-7, respectively. Recovery is determined if patients transitioned from being at caseness pre-treatment to non-caseness post-treatment, where caseness is defined as scores ≥ 10 on the PHQ-9 and ≥ 8 on the GAD-7. Reliable Improvement is determined when there is a decrease in the PHQ-9 or the GAD-7 that is greater than the RCI and with no increase on either measure larger than the RCI. Finally, Reliable Recovery is determined when patients achieved both Recovery and Reliable Improvement.

To investigate whether the interventions were overall more effective in improving clinical outcomes in patients with or without LTC, analyses of covariance (ANCOVAs) were used, with each clinical outcome serving as dependent variables, time (pre–post-treatment) and LTC status (LTC or non-LTC) as independent variables, and baseline severity of clinical scores as a covariate. To determine which, if any, of the low-intensity interventions were associated with comparatively greater changes in clinical outcomes, and whether there is a differential pattern of intervention effectiveness for LTC and non-LTC patients, the above analyses were repeated with the addition of intervention (iCBT, PGT, GSH) as an independent variable in the models. Additionally, control analyses on intervention-specific effects were conducted with the LTC cohort specifically. Considering imbalances in age and gender ratios within each cohort across the interventions (see Table 1), the models were controlled for potential effects of age and gender by including them as covariates. All potential interactions between independent variables were modelled, and Tukey adjusted pairwise comparisons were conducted to decompose significant interaction effects. Data analyses were carried out using R statistical package Version 4.1.1.

**Table 1:** Sample characteristics of LTC and non-LTC cohorts across intervention groups (iCBT, PGT, GSH)

	**LTC status group comparisons**			
**Characteristics**	**LTC status**		**χ2 / t (df)**	***p***
	LTC (*n* = 4024)	Non-LTC (*n* = 17,027)		
**Gender **(*N*, %)			2.10 (1)	0.147
Female	2620 (65.10)	11,291 (66.31)		
Male	1404 (34.90)	5736 (33.69)		
**Age**				
Mean, SD (range)	46.05, 15.88 (18–80)	35.79, 12.96 (18–80)	−43.14 (21,049)	< 0.001***
**Baseline PHQ-9**				
Mean, SD	14.46 (6.03)	13.71 (5.85)	−7.31 (21,049)	< 0.001***
**Baseline GAD-7**				
Mean, SD	12.82 (5.17)	12.98 (4.86)	1.89 (21,049)	0.059
**Baseline WSAS**				
Mean, SD	17.83 (9.70)	16.80 (8.88)	−6.52 (21,049)	< 0.001***
**Treatment duration (days)**^**a**^				
Mean, SD	90.47 (58.25)	89.68 (53.63)	−0.75 (17,431)	0.452
**Number of appointments**				
Mean, SD	5.14 (2.64)	5.10 (2.41)	−0.91 (21,049)	0.361
**IAPT clinical outcomes**				
** Caseness** *N* (%)	3636 (90.36)	15,516 (91.13)	2.34 (1)	0.126
** Recovery** (*N*, %)	1984 (54.57)	8618 (55.54)	1.10 (1)	0.295
** Reliable improvement** (*N*, %)	2364 (65.02)	10,334 (66.60)	3.24 (1)	0.072
** Reliable recovery** (*N*, %)	1803 (49.59)	7848 (50.58)	1.12 (1)	0.290

^a^Instances where treatment duration is 0 due to administrative errors were removed from analyses

## Results

Of the 21,051 patients that were included in the study, 12,746 received GSH, 6857 received iCBT, and 1448 received PGT. Amongst the 4024 patients that had at least one LTC, 2620 patients underwent GSH, 1065 patients underwent iCBT, and 339 patients underwent PGT. Table 1 outlines the demographic and clinical characteristics of both samples. The gender ratio for both LTC patients (65.1% females) and non-LTC patients (66.3% females) were comparable, *χ*2_Gender_ (1) = 2.10, *p* = 0.15. However, the LTC cohort (*M* = 46.05, SD = 15.88) were on average older than those without a LTC (*M* = 35.79, SD = 12.96), *t*(21,049) =  − 43.14, *p* < 0.001. In relation to pre-treatment symptom severity, the LTC cohort (*M* = 14.46, SD = 6.03) had significantly higher levels of depression than their non-LTC counterparts (*M* = 13.71, SD = 5.85), *t*(21,049) =  − 7.31, *p* < 0.001. Similarly, the LTC patients also had significantly higher pre-treatment functional impairment (*M* = 17.83, SD = 9.70) than non-LTC patients (*M* = 16.80, SD = 8.88), *t*(21,049) =  − 6.52, *p* < 0.001. Anxiety scores were not significantly higher in the non-LTC cohort (*M* = 12.98, SD = 4.86) than in the LTC cohort (*M* = 12.82, SD = 5.17), though there was a trend towards significance, *t*(21,049) = 1.89, *p* = 0.059. The two cohorts did not differ in the mean number of treatment days (*p* = 0.452) nor in the number of clinician-guided appointments or reviews (*p* = 0.361) (see Supplementary Materials 1 and 2 for additional demographics and treatment characteristics).

### Clinically Significant Changes

For both the LTC and non-LTC cohorts, around 90% of patients met the criteria for caseness at pre-treatment (LTC *n* = 3,636, 90.36%, non-LTC *n* = 15,516, 91.13%, *χ*2_Caseness_ (1) = 2.34, *p* = 0.126). Over half of both cohorts achieved recovery (LTC *n* = 1984, 54.57%, non-LTC *n* = 8618, 55.54%, χ2_Recovery_ (1) = 1.10, *p* = 0.295), and around two-thirds of both cohorts achieved reliable improvement (LTC *n* = 2364, 65.02%, non-LTC *n* = 10,334, 66.60%, χ2_Reliable improvement_ (1) = 3.24, *p* = 0.072). The proportion of patients who achieved reliable recovery in the LTC cohort (*N* = 1803, 49.59%) and the non-LTC cohort (*N* = 7848, 50.58%), which is approximately half for both samples, were also comparable, χ2_Reliable recovery_ (1) = 1.12, *p* = 0.290 (see Table 1).

### Overall Intervention Effectiveness for LTC vs. Non-LTC Patients

**Fig. 1:**
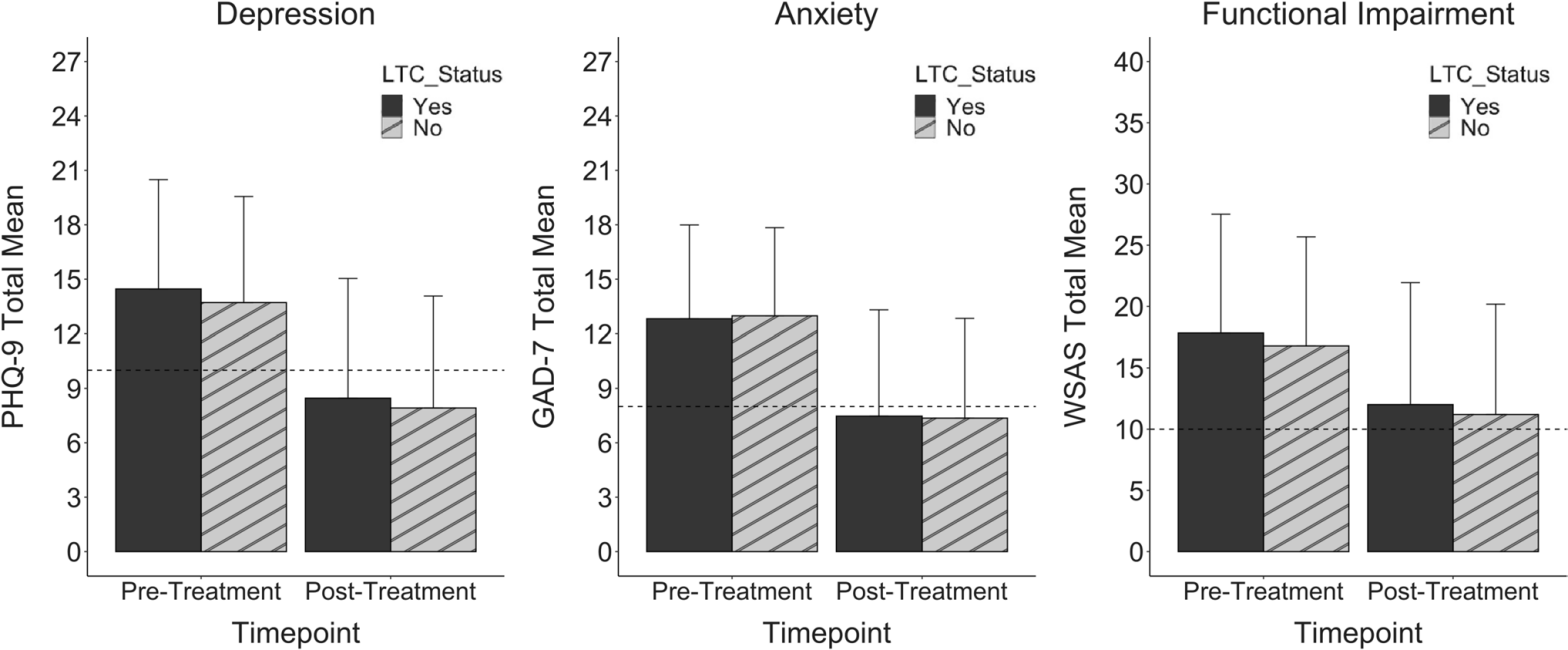
Observed means of LTC and non-LTC service users at pre-treatment and post-treatment for each outcome measure. Note. The dashed line indicates the threshold for caseness for each measure and the error bars indicate standard deviation of the mean. LTC patients showed slightly greater reductions in depression symptoms relative to non-LTC patients, while non-LTC patients showed slightly greater reductions in anxiety symptoms when compared to their LTC counterpart. There were no group differences in the extent to which functional impairment improved post-treatment

A comparison of intervention effects between the LTC and non-LTC cohort was conducted using two-way ANCOVAs with baseline severity as a covariate (see Fig. 1). For depression, there was a general reduction in PHQ-9 scores from pre- to post-treatment, *F*(1, 42097) = 13551.39, *p* < 0.001. A significant LTC status by time interaction was also evident, *F*(1, 42097) = 4.31, *p* = 0.038, driven by the LTC cohort experiencing a greater reduction in symptoms (adj. pre M = 14.00, SE = 0.06; adj. post M = 8.00, SE = 0.06, *d* = 1.47) than the non-LTC cohort (adj. pre M = 13.82, SE = 0.03; adj. post M = 8.00, SE = 0.03, *d* = 1.42). For anxiety, GAD-7 scores overall reduced from pre- to post-treatment, *F*(1, 42097) = 13645.64, *p* < 0.001. While there was also a significant time by LTC status interaction, *F*(1, 42097) = 8.70, *p* = 0.003, in contrast to depression, reductions in anxiety were also greater for the non-LTC cohort (adj. pre *M* = 12.96, SE = 0.03; adj. post *M* = 7.34, SE = 0.03, *d* = 1.48) compared to the LTC cohort (adj. pre *M* = 12.91, SE = 0.06; adj. post *M* = 7.57, SE = 0.06, *d* = 1.41). In terms of functional impairment, WSAS scores also reduced from pre- to post-treatment, *F*(1, 42097) = 5602.91, *p* < 0.001, but having a LTC did not influence this, *F*(1, 42097) = 2.18, *p* = 0.140. Table 2 provides an overview of the adjusted means for all three outcome measures at pre- and post-treatment for LTC and non-LTC patients.

**Table 2:** Adjusted means of each outcome measure for LTC and non-LTC service users at pre-treatment (pre) and post-treatment (post)

Measures	LTC	Non-LTC	F (df)	p
	Pre v. Post M (SE)	Pre v. Post M (SE)		
PHQ-9	14.00 (0.06) v. 8.00 (0.06)	13.82 (0.03) v. 8.03 (0.03)	4.31 (1, 42097)	0.038*
GAD-7	12.91 (0.06) v. 7.57 (0.06)	12.96 (0.03) v. 7.33 (0.03)	8.70 (1, 42097)	< 0.001***
WSAS	17.21 (0.10) v. 11.39 (0.10)	16.94 (0.05) v. 11.35 (0.05)	2.18 (1, 42097)	0.140

*GAD-7* Generalised Anxiety Disorder-7 item questionnaire, *LTC* long-term condition, *PHQ-9* Patient Health Questionnaire-9 item, *WSAS* work and social adjustment scale, *SE* standard error

### Intervention-Specific Effectiveness for LTC vs. Non-LTC Patients

**Fig. 2:**
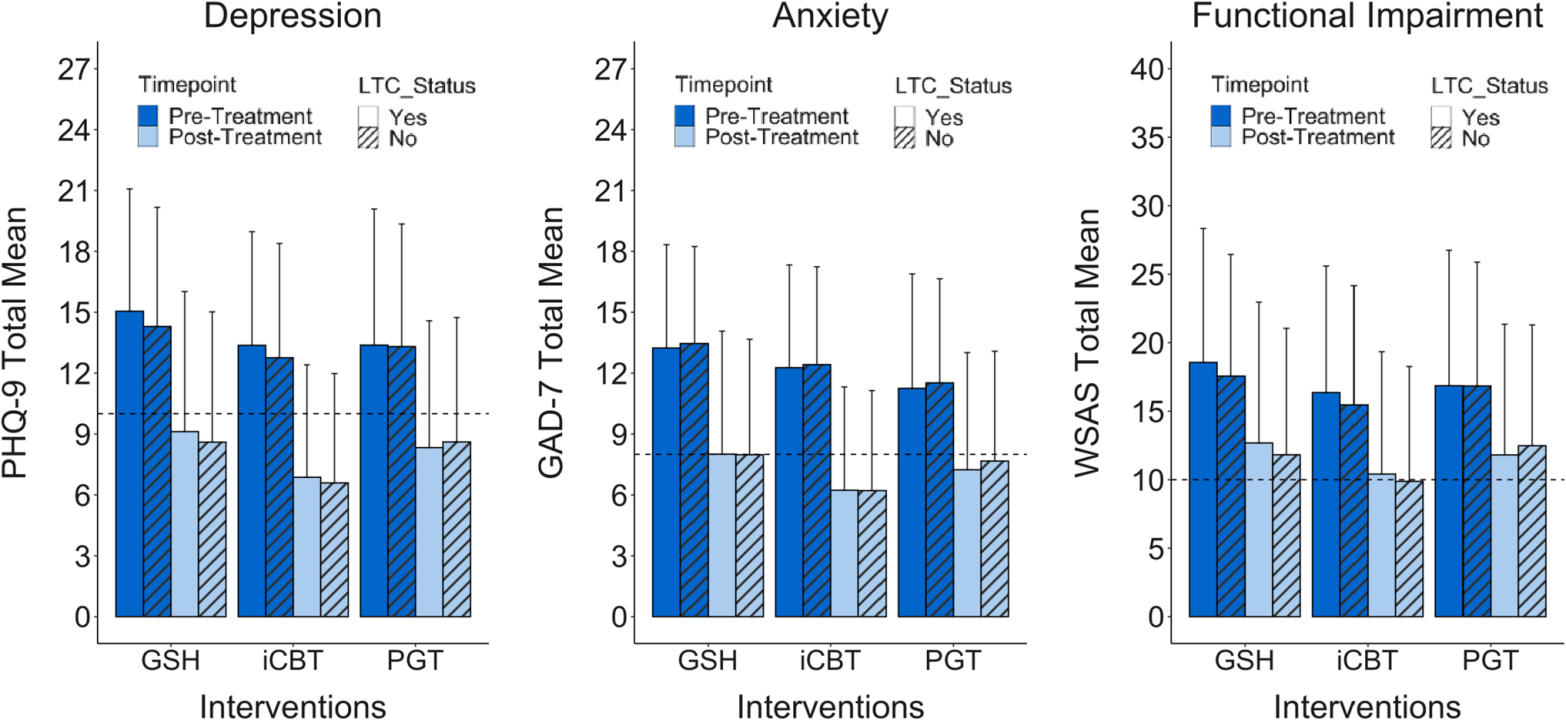
Pre-treatment to post-treatment observed means in depression, anxiety, and functional impairment exhibited by LTC and non-LTC patients across GSH, iCBT, and PGT interventions. Note. The dashed line indicates the threshold for caseness for each measure and the error bars indicate standard deviation of the mean. For depression and anxiety symptoms, iCBT was associated with the greatest improvements, followed by GSH, and then PGT. For functional impairment, both iCBT and GSH were associated with greater improvement than PGT. For all clinical outcomes, the effectiveness of interventions is consistent across LTC and non-LTC patients

Three-way ANCOVAs were employed to investigate differences in the effectiveness of specific low-intensity psychological interventions (iCBT, GSH, and PGT) for LTC vs non-LTC patients, with baseline severity as a covariate (see Fig. 2). For depression, a significant time by intervention interaction indicated decreases in PHQ-9 scores according to intervention type, *F*(2, 42089) = 27.52, *p* < 0.001. Post hoc tests indicated that iCBT was associated with the greatest improvements in PHQ-9 score (adj. pre *M* = 13.66, SE = 0.07; adj. post *M* = 7.33, SE = 0.07, *d* = 1.55), compared to GSH (adj. pre *M* = 14.06, SE = 0.04; adj. post *M* = 8.24, SE = 0.04, *d* = 1.43) and PGT (adj. pre *M* = 13.73, SE = 0.13; adj. post *M* = 8.86, SE = 0.13, *d* = 1.20), while those in GSH improved more than those in PGT (all *p* < 0.001). However, there was no three-way interaction, *F*(2, 42089) = 0.14, *p* = 0.87, suggesting that the effectiveness of these treatments is consistent across LTC and non-LTC patients. Likewise, for anxiety, there was no significant three-way interaction between time, intervention, and LTC status, *F*(2, 42089) = 0.65, *p* = 0.52. As with depression, there was a significant interaction between time and intervention, *F*(2, 42089) = 72.46, *p* < 0.001, where iCBT was associated with the greatest improvement in GAD-7 scores (adj. pre *M* = 12.79, SE = 0.06; adj. post *M* = 6.66, SE = 0.06, *d* = 1.63), followed by GSH (adj. pre *M* = 13.06, SE = 0.04; adj. post *M* = 7.70, SE = 0.04, *d* = 1.42) and PGT (adj. pre *M* = 12.53, SE = 0.12; adj. post *M* = 8.60, SE = 0.12, *d* = 1.04). Similarly for functional impairment, there was no three-way interaction between time, intervention, and LTC status, F(2, 42089) = 0.60, *p* = 0.55, but a significant time by intervention interaction suggested that the interventions were associated with different degrees of improvement, *F*(2, 42089) = 7.55, *p* < 0.001. While patients in both GSH (adj. pre *M* = 17.30, SE = 0.07; adj. post *M* = 11.50, SE = 0.07, *d* = 0.94) and iCBT (adj. pre *M* = 16.70, SE = 0.10; adj. post *M* = 10.90, SE = 0.10, *d* = 0.94) improved more in functioning than those in PGT (adj. pre *M* = 17.00, SE = 0.19; adj. post *M* = 12.30, SE = 0.19, *d* = 0.77) (all *p* < 0.001), there were no group differences between patients in GSH and iCBT (*p* = 0.816). Table 3 provides an overview of the adjusted means for each clinical outcome at pre- and post-treatment for all patients across each of the interventions.

**Table 3:** Adjusted means of each outcome measure for patients across intervention groups at pre-treatment (pre) and post-treatment (post)

Measures	GSH	iCBT	PGT	*F* (df)	*p*
	Pre v. Post M (SE)	Pre v. Post M (SE)	Pre v. Post M (SE)		
PHQ-9	14.06 (0.04) v. 8.24 (0.04)	13.66 (0.07) v. 7.33 (0.07)	13.73 (0.13) v. 8.86 (0.13)	27.52 (2, 42089)	< 0.001***
GAD-7	13.06 (0.04) v. 7.70 (0.04)	12.79 (0.06) v. 6.67 (0.06)	12.53 (0.12) v. 8.60 (0.12)	72.46 (2, 42089)	< 0.001***
WSAS	17.27 (0.07) v. 11.47 (0.07)	16.71 (0.10) v. 10.95 (0.10)	16.96 (0.19) v. 12.25 (0.19)	7.55 (2, 42089)	< 0.001***

*GAD-7* Generalised Anxiety Disorder-7 item questionnaire, *LTC* long-term condition, *PHQ-9* Patient Health Questionnaire-9 item, *WSAS* work and social adjustment scale, *SE* standard error

### Control Analyses

To explore whether age and gender explained any of the observed effects, these variables were added as covariates to the models for each outcome measure. There was a significant main effect of age in each model (all *p* < 0.001), indicating that older age was generally associated with less severe symptoms. However, each of the observed time by intervention interactions remained significant, suggesting that age and gender did not explain the observed effects in the models. Additional analyses investigating intervention-specific effectiveness in only the LTC cohort revealed similar patterns as the above analyses (see Supplementary Materials 3).

## Discussion

This study investigated the effectiveness of low-intensity psychological interventions for treating comorbid depression and anxiety in LTC patients through retrospective analyses of large-scale patient data from a real-world mental health service. As these low-cost, scalable interventions become more mainstream in mental healthcare services, it is important to establish their therapeutic benefit in new cohorts, tested in real-world contexts. The study found that LTC patients presented with varying demographics and clinical characteristics compared to their non-LTC counterparts (i.e. LTC patients were older, more depressed, and more functionally impaired). Accounting for gender, age, and symptom severity differences, statistically significant differences were observed in the way LTC and non-LTC patients improved from low-intensity psychological interventions, where LTC patients yielded greater reduction in depression symptoms and non-LTC patients yielded greater reduction in anxiety symptoms. However, it is important to note that the overall effect sizes of these treatments were large for both cohorts (all Cohen’s *d* > 1.4), with marginal differences between them (i.e. Cohen’s *d* differences < 0.08) that amounted to < 1 score difference on both depression and anxiety measures post-treatment. The aim of CBT-based interventions is to address specific mental health issues. In line with this, the low-intensity psychological interventions employed here did exactly what they were purposed to do, which is to treat the symptoms of depression and anxiety regardless of LTC status. This observation of a general improvement in depression, anxiety, and functional impairment irrespective of LTC status constitutes some of the first evidence from large-scale real-world patient data to support the use of these interventions to treat comorbid anxiety and depression in LTC patients.

Consistent with the above, there was no effect of LTC status when investigating the differential effectiveness of three low-intensity psychological interventions for LTC and non-LTC patients. While all interventions were shown beneficial, iCBT was found to be the most effective in reducing both depression and anxiety symptoms, relative to PGT and GSH, for both cohorts. A similar pattern of results were observed when the LTC cohort was examined specifically. Overall, the finding that iCBT is generally more effective than GSH and PGT for reducing depression and anxiety in general patients enrolled in IAPT has been reported elsewhere [14]. We thus focus on discussing the relevance and implication of our results for LTC patients. iCBT employs the same principles and components as traditional face-to-face CBT, with many additional benefits that may prove particularly beneficial for the LTC cohort. Murray (2008) states that the combination of online psychoeducation with clinician support allows patients to interpret and internalise available information. This can lead to change in and interaction between knowledge motivation, emotional state, and self-efficacy for improved health behaviours and in turn improve LTC-related outcomes (e.g. understanding risks may alter anxiety which can lead to enhanced motivation to improve health) [27]. Furthermore, by integrating technological advances into the treatment approach, iCBT bypasses the need for in-person attendance by presenting an online solution that is stigma-free and easily accessible, which can facilitate LTC patients’ access to healthcare due to their limited physical capacity [28]. In relation to GSH, LTC patients may also find its remote service delivery to be beneficial in terms of increased flexibility and accessibility. While clinician support in GSH may not be as frequent or readily available as that in iCBT, research has suggested adding an element of guided support performs better than no support [29, 30]. In this regard, while one may argue PGT involves the most intensive form of clinician support as it is delivered and facilitated in a face-to-face format, it requires LTC patients to attend in-person, thus hindering the real-world accessibility of this intervention to those with physical limitations within this cohort.

Furthermore, our findings revealed that gender and age did not influence the effectiveness of each intervention, but rather, less severe clinical symptoms were observed in older patients. This is in line with a broader body of research highlighting the association between aging and decreased susceptibility to depression and anxiety [31] which may be partially due to an accumulation of buffering psychosocial protective factors across the lifespan [32]. Within the context of chronic illness, younger persons with LTCs may potentially experience greater disruption to identity and routine life events than their older counterparts, which may lead to elevated levels of psychological difficulties [33, 34].

According to Lorig and Holman (2003), the use of CBT constitutes a vital component of LTC management in addressing the emotional difficulties accompanying the condition [35]. The clinically significant improvements in depression and anxiety exhibited by real-world LTC patients in the present study thus builds upon existing research where prior studies and trials have shown the effectiveness of CBT-based interventions for treating comorbid depression and anxiety across a variety of LTCs [36–39]. Furthermore, improvements in functioning amongst LTC patients was also observed post-treatment, which is in line with previous studies highlighting the utility of psychological interventions for improving the impact LTCs can have on one’s quality of life, routine functioning, and wellbeing [40]. All in all, CBT assists LTC patients in gaining a better understanding of their illness by increasing their awareness of negative thoughts and unhelpful behaviours, while allowing them to become active participants in their own wellbeing journey through the development of adequate self-management mechanisms [40].

### Strengths and Limitations

Several strengths and limitations of the present study must be considered. The study entailed a real-world analysis of a large, naturalistic patient sample enrolled in routine care, thus underscoring the ecological validity of our findings. While the generalisability of our results beyond the service within which this study has been conducted may be limited, patients were recruited from a representative mental health service in Berkshire NHS Trust, UK, of which outcome data is suggested to be comparable to the nationwide data. Nevertheless, given the nature of our retrospective, observational analyses, there may exist biases in treatment allocation that could potentially affect our results (e.g. selection bias in decision-making by clinicians and patients). There were also a lack of differences in the overall and intervention-specific treatment effects between LTC and non-LTC patients, thus warranting caution in over-interpreting the specificity of the order of treatment effects for the LTC cohort. Furthermore, both LTC and non-LTC cohorts in our study experienced, on average, moderate depression and/or anxiety symptomatology for which the low-intensity psychological interventions were intended. Thus, we cannot comment on the effectiveness of these low-intensity interventions for LTC patients who experience more severe depression and/or anxiety and may require more intensive care. Finally, a considerable weakness of the study refers to the lack of information regarding the specific chronic illness of each LTC patient (e.g. type and duration of the LTC, number of comorbidities). This was not available to the authors for analyses in the study, and as such, this study could only shed light on the effectiveness of low-intensity psychological interventions on LTC as a whole, whereas treatment effects may differ dependent on the type of chronic illness. Our study also lacked data from disorder-specific measures. It may be that accounting for pre-treatment disorder-specific distress levels would influence the intervention effects.

### Implications

Comorbid depression and anxiety in patients with LTC pose grave implications to the prognosis of the patient, while increasing the burden on healthcare systems to meet the demands of mental health services. Low-intensity psychological interventions are available to help alleviate this burden. Findings of this study underscore the effectiveness of these interventions, in particular iCBT, in treating comorbid depression and anxiety in LTC patients. This has potential implications for informing decision-making regarding treatment allocation for this particular cohort. Our study further supports the delivery of mental health services via integrated care and contributes to the literature highlighting many positive effects brought about by the model, including patient satisfaction, improved access, and increased perceived quality of care [41] as well as improved control and reporting of clinical outcomes [42]. Beyond the IAPT programme in the UK, the successful implementation of integrated care models has also been observed in other countries including Canada, the US, and Australia [41].

### Future Research

Stemming from our approach, future research should expand the scope by examining real-world data from multiple, geographically different IAPT services to further increase the generalisability of our findings. Comparing different services across the UK would also help identify and control for confounding covariates. While our results demonstrated no clinically significant differences in the way low-intensity psychological interventions work for LTC and non-LTC patients, recent evidence has suggested that generic CBT-based interventions might fail to acknowledge the role of LTC in mental illness. However, tailored interventions that consider the complex interactions between the physical and mental health conditions may make treatments more meaningful and relevant for the LTC cohort [43]. Future research could thus examine the extent to which tailored versus standardised low-intensity CBT-based interventions in treating comorbid depression and anxiety in LTC patients, so to verify whether the personalised version of the interventions results in greater improvements. Future research should also consider including disorder-specific measures, such as the diabetes-distress scale [44], as these are likely more capable of capturing the particular psychological distress associated with each chronic illness. These measures would also likely be more sensitive to tailored interventions, and significant improvement on disease-specific outcomes could in turn provide justification for tailoring interventions to specific cohorts [28]. Another point of interest for future research regarding iCBT is its cost-effectiveness. The fact that iCBT can reduce significant public and individual costs can be hugely beneficial [45]. Evidence-based research on this key concept of health economics with particular consideration of the burdens associated with LTC is warranted to inform the wider implementation of iCBT in healthcare systems at both national and international levels.

## Conclusion

With long-term conditions exerting a life-changing impact on an individual’s wellbeing and functioning, it is vital to have interventions in place that are effective in mitigating the psychological comorbidities accompanying these physical illnesses. The current study is the first to highlight the effectiveness of low-intensity CBT interventions for treating comorbid, clinically significant depression and anxiety symptoms in people with LTCs in a naturalistic routine care setting. The treatment effects demonstrated in this study support the implementation of low-intensity psychological interventions, particularly iCBT, as effective treatments for LTC patients. Further extension of iCBT as a treatment for depression and anxiety in people with LTC will alleviate the burden on healthcare systems by meeting the increasing demands for mental health services amongst this population.

## Supplementary Information

Below is the link to the electronic supplementary material.

Supplementary file1 (DOCX 32 KB)

## Data Availability

De-identified data of this study are not available in a public archive, however, the analytic codes and materials of this study are available upon reasonable request.
