# Correction: The Effectiveness of Low-Intensity Psychological Interventions for Comorbid Depression and Anxiety in Patients with Long-Term Conditions: A Real-World Naturalistic Observational Study in IAPT Integrated Care

**DOI:** 10.1007/s12529-023-10226-6

**Published:** 2023-10-17

**Authors:** Chi Tak Lee, Siobhan Harty, Adedeji Adegoke, Jorge Palacios, Claire M. Gillan, Derek Richards

**Affiliations:** 1 School of Psychology, Trinity College Dublin, Dublin, Ireland; 2 Trinity College Institute of Neuroscience, Trinity College Dublin, Dublin, Ireland; 3 Global Brain Health Institute, Trinity College Dublin, Dublin, Ireland; 4 SilverCloud Science, SilverCloud Health, Dublin, Ireland

**Correction to: International Journal of Behavioral Medicine** **https://doi.org/10.1007/s12529-023-10215-9**

The original online version of this article was revised: The author Jorge Palacios is affiliated with both SilverCloud Science, SilverCloud Health, Dublin, Ireland and School of Psychology, Trinity College Dublin, Dublin, Ireland. 

The original article has been corrected.

