# Supplementary material for: The Effectiveness of Low-Intensity Psychological Interventions for Comorbid Depression and Anxiety in Patients with Long-Term Conditions: A Real-World Naturalistic Observational Study in IAPT Integrated Care
Source: Int J Behav Med. 2023 Sep 11;31(5):730–40. doi: 10.1007/s12529-023-10215-9 (PMC11452547; doi:10.1007/s12529-023-10215-9)
Supplement: Supplementary file 1 — Supplementary file1 (DOCX 32 KB) [file 12529_2023_10215_MOESM1_ESM.docx]

**Supplementary Materials**

**Table of Contents**

[Supplementary Material 1 1](#_Toc135653194)

[Supplementary Material 2 3](#_Toc135653195)

[Supplementary Material 3 4](#_Toc135653196)

# **Supplementary Material 1**

**Table S1.**

***Comprehensive sample characteristics of LTC and non-LTC cohorts across intervention groups (iCBT, PGT, GSH).***

| **LTC Status Group Comparisons** |  | |  |  |
| --- | --- | --- | --- | --- |
| **Characteristics** | **LTC Status** | | **χ2 / t (df)** | **p** |
|  | LTC (n=4024) | Non-LTC (n=17027) |  |  |
| **Gender (N, %)**  Female  Male | 2620 (65.10)  1404 (34.90) | 11,291 (66.31)  5736 (33.69) | 2.10 (1) | 0.147 |
| **Age**  Mean, SD (Range) | 46.05, 15.88 (18-80) | 35.79, 12.96 (18-80) | -43.14 (21049) | < 0.001*** |
| **Employment (N, %)^a^**  Employed  Unemployed  Student  Retired  Other | 2385 (60.04)  447 (11.30)  131 (3.32)  563 (14.20)  424 (10.70) | 12602 (75.20)  1457 (8.56)  1143 (6.83)  575 (3.43)  969 (5.79) | 982.02 (4) | < 0.001*** |
| **Ethnicity (N, %)^b^**  British  Other White Background  Asian  Caribbean  African  Other | 3038 (78.70)  179 (4.64)  391 (10.10)  85 (2.20)  55 (1.42)  113 (2.93) | 12866 (77.90)  1172 (7.10)  1460 (8.84)  327 (1.98)  207 (1.25)  477 (2.89) | 36.47 (6) | < 0.001*** |
| **Baseline PHQ-9**  Mean, SD | 14.46 (6.03) | 13.71 (5.85) | -7.31 (21049) | < 0.001*** |
| **Baseline GAD-7**  Mean, SD | 12.82 (5.17) | 12.98 (4.86) | 1.89 (21049) | 0.059 |
| **Baseline WSAS**  Mean, SD | 17.83 (9.70) | 16.80 (8.88) | -6.52 (21049) | < 0.001*** |
| **Treatment Duration (Days)^c^**  Mean, SD | 90.47 (58.25) | 89.68 (53.63) | -0.75 (17431) | 0.452 |
| **Number of Appointments**  Mean, SD | 5.14 (2.64) | 5.10 (2.41) | -0.91 (21049) | 0.361 |
| **IAPT Clinical Outcomes** |  |  |  |  |
| **Caseness** N (%) | 3636 (90.36) | 15516 (91.13) | 2.34 (1) | 0.126 |
| **Recovery** (N, %) | 1984 (54.57) | 8618 (55.54) | 1.10 (1) | 0.295 |
| **Reliable Improvement** (N, %) | 2364 (65.02) | 10334 (66.60) | 3.24 (1) | 0.072 |
| **Reliable Recovery** (N, %) | 1803 (49.59) | 7848 (50.58) | 1.12 (1) | 0.290 |

^a^N = 355 missing data for employment information. ^b^N = 681 missing data for ethnicity information. ^c^Instances where treatment duration is 0 due to administrative errors were removed from analyses.

# **Supplementary Material 2**

**Table S2.**

***Treatment characteristics of LTC and non-LTC cohorts within iCBT intervention group***

| **Characteristics** | **LTC Status** | | **t (df)** | **p** |
| --- | --- | --- | --- | --- |
|  | LTC (n=1065) | Non-LTC (n=5792) |  |  |
| **Treatment Duration (Days)^a^**  Mean, SD | 93.93 (53.03) | 90.19 (49.35) | -2.18 (6855) | 0.029* |
| **Number of Appointments**  Mean, SD | 5.63 (2.34) | 5.54 (2.22) | -1.13 (6855) | 0.257 |

^a^Instances where treatment duration is 0 due to administrative errors were removed from analyses (N=427)

**Table S3.**

***Treatment characteristics of LTC and non-LTC cohorts within PGT intervention group***

| **Characteristics** | **LTC Status** | | **t (df)** | **p** |
| --- | --- | --- | --- | --- |
|  | LTC (n=339) | Non-LTC (n=1109) |  |  |
| **Treatment Duration (Days)^a^**  Mean, SD | 52.34 (48.50) | 48.48 (38.11) | -1.52 (1446) | 0.130 |
| **Number of Appointments**  Mean, SD | 4.92 (2.55) | 4.68 (2.08) | -1.80 (1446) | 0.072 |

^a^Instances where treatment duration is 0 due to administrative errors were removed from analyses (N=13).

**Table S4.**

***Treatment characteristics of LTC and non-LTC cohorts within GSH intervention group***

| **Characteristics** | **LTC Status** | | **t (df)** | **p** |
| --- | --- | --- | --- | --- |
|  | LTC (n=2620) | Non-LTC (n=10126) |  |  |
| **Treatment Duration (Days)^a^**  Mean, SD | 95.07 (59.93) | 95.32 (55.86) | 0.18 (12744) | 0.860 |
| **Number of Appointments**  Mean, SD | 4.97 (2.74) | 4.89 (2.51) | -1.36 (12744) | 0.173 |

^a^Instances where treatment duration is 0 due to administrative errors were removed from analyses (N=3,178).

# **Supplementary Material 3**

**Intervention-Specific Effectiveness Analyses for LTC patients**

For depression, there was a significant main effect for time, indicating that the LTC cohort experienced an overall reduction in PHQ-9 scores from pre-treatment to post-treatment, F(1, 8041) = 2029.52, p < 0.001. The extent to which LTC patients reduced their PHQ-9 scores from pre-treatment to post-treatment varied across intervention type, as evidenced by a time by intervention group significant interaction, F(2, 8041) = 8.37, p < 0.001. Results from Tukey post-hoc tests indicated that LTC patients engaged in iCBT exhibited significantly greater improvements in PHQ-9 score (adj. pre-treatment M = 14.22, SE = 0.13; adj. post-treatment M = 7.72, SE = 0.13, d = 1.55) than those in GSH (adj. pre-treatment M = 14.60, SE = 0.08; adj. post-treatment M = 8.67, SE = 0.08, d = 1.41, p = 0.008) and PGT (adj. pre-treatment M = 14.22, SE = 0.23; adj. post-treatment M = 9.17, SE = 0.23, d = 1.20, p < 0.001), while GSH was associated with greater improvements than PGT (p = 0.010). A similar pattern was observed for anxiety measured on the GAD-7; a significant main effect of time indicated an overall reduction in GAD-7 scores experienced by the LTC cohort, F(1, 8041) = 1832.88, p < 0.001. A significant interaction effect between time and intervention indicated that LTC patients across each intervention differed from each other in the extent their anxiety symptoms improved, F(2, 8041) = 19.22, p < 0.001. LTC patients in iCBT exhibited greater improvement in GAD-7 scores (adj. pre-treatment M = 12.68, SE = 0.12; adj. post-treatment M = 6.65, SE = 0.12, d = 1.56) when compared to PGT (adj. pre-treatment M = 12.43, SE = 0.21; adj. post-treatment M = 8.43, SE = 0.21, d = 1.04, p < 0.001) and GSH (adj. pre-treatment M = 12.92, SE = 0.08; adj. post-treatment M = 7.68, SE = 0.08, d = 1.36, p = 0.001), while those in GSH improved more than those in PGT (p = 0.001). For functional impairment, there was a significant main effect of time, F(1, 8041) = 822.74, p < 0.001, reflecting a general reduction in functional impairment from pre-treatment to post-treatment. However, there was no significant interaction effect between time and intervention group, indicating that reductions in impairment scores did not vary across the intervention groups, F(2, 8041) = 1.40, p = 0.247.

To explore whether sociodemographic differences such as age and gender of the LTC patients explained any of the observed interaction effects, we added age and gender as covariates to the models for each outcome measure. There was a significant main effect of age in each model (all p < 0.001), indicating that older age was generally associated with less severe symptoms. However, each of the observed time by intervention type interaction effects remained significant. Thus, neither age or gender explained the superiority of iCBT over GSH and PGT, nor the superiority of GSH over PGT, for reducing anxiety and depression symptoms in LTC patients.
